# The association between use of social media and the development of body dysmorphic disorder and attitudes toward cosmetic surgeries: a national survey

**DOI:** 10.3389/fpubh.2024.1324092

**Published:** 2024-03-08

**Authors:** Khadijah Ateq, Mohammed Alhajji, Noara Alhusseini

**Affiliations:** ^1^Department of Biostatistics, Epidemiology and Public Health, College of Medicine, Alfaisal University, Riyadh, Saudi Arabia; ^2^Research Office, King Abdullah International Medical Research Center (KAIMRC), King Saud Bin Abdulaziz University for Health Science (KSAU-HS), Riyadh, Saudi Arabia; ^3^Behavioral Insight Unit, Ministry of Health, Riyadh, Saudi Arabia

**Keywords:** body dysmorphic disorder, cosmetic surgery, mental health, social media, Snapchat, Instagram, selfies, filters

## Abstract

**Introduction:**

Body dysmorphic disorder (BDD) causes distress due to one's negative appraisal of their body image. The development of BDD has been linked to the passive use of social media and photo-editing apps. People with BDD typically pursue cosmetic surgeries to remedy their perceived flaws. The dramatic increase in the use of photo-editing apps and their well-established effects on mental health is a public health concern.

**Purpose:**

To study the association between use of social media and the development of BDD and acceptance toward cosmetic surgeries (ACSS) among Saudis.

**Methods:**

An online, cross-sectional, validated survey conducted among Saudis 18 and older. Descriptive analyses were utilized for demographics and prevalence rates of main study variables. ANOVA was used to compare mean scores in BDD and ACSS among different demographic groups. Tukeys *post-hoc* test was done to identify the categories that were different when the ANOVA test showed a statistically significance. A *p*-value of <0.05 was considered statistically significant.

**Results:**

A total of 1,483 Saudi adults completed the questionnaire. Key results showed that BDD was found in 24.4 % of the sample. The percentage of participants with BDD who spent 4–7 h per day on Instagram and Snapchat (29%) was significantly higher than those who spent only less than an hour per day on these platforms (19%) (*p* < 0.001). Individuals with BDD had a significantly higher risk of accepting cosmetic surgery compared to those without BDD (*p* < 0.001).

**Conclusion:**

A growing body of evidence suggests that social media may impact mental health in different ways. This study reveals that heavy use of these platforms is associated with negative appraisals about one's physical appearance, and it fosters one's tendency toward cosmetic surgery, especially among females.

## Introduction

### Body dysmorphic disorder prevalence

Body Dysmorphic Disorder (BDD) has received considerable attention in the last two decades, and it is widely regarded as one of the most debilitating mental health conditions (1–3). BDD is a chronic psychiatric disorder recognized by excessive preoccupation with minimal physical deformities (1, 4). Such preoccupations can cause excessive thinking and compulsive behaviors, which may impede everyday life (5). According to the Diagnostic and Statistical Manual of Mental Disorders-Fourth Edition (DSM-IV), BDD is defined as an obsession with an actual or perceived minor flaw in one's appearance (6). BDD is considered an obsessive-compulsive disorder (OCD) by the American Psychiatric Association (APA) and the Diagnostic and Statistical Manual of Mental Disorders- Fifth Edition (DSM-V) (6). The second diagnostic criterion for BDD is significant stress or emotional suffering, including depression, sadness, worry, anxiety, and other negative thoughts or feelings (1). Finally, for a diagnosis to be considered BDD, another mental illness should not explain the patient's preoccupation, such as anorexia nervosa and dissatisfaction with body shape (1).

Although diagnostic criteria exist, BDD is frequently misdiagnosed and underreported (1). A recent survey shows that only 15% of people with BDD are actually diagnosed (3). Even when people with BDD present themselves for mental health support, BDD symptoms are frequently missed or misdiagnosed with other illnesses, such as depression or social anxiety (3). Another reason why some BDD may have gone unnoticed is that people with BDD frequently mask their body image concerns out of shame or embarrassment or are dismissed (3). In recent estimates in Saudi Arabia, BDD is estimated to occur in 1.9 % of adults (1). BDD is expected to affect up to 1% of the U.S. population (2). A systematic review of BDD prevalence results the general population (0.5–3.2%). Also, this number is expected to be underestimated due to the reality that people with BDD feel stigmatized and do not seek help (7). Further, population-based studies show alarmingly high rates of suicidality in youth with BDD, which emphasizes the clinical importance of BDD (1, 3).

Patients with BDD frequently feel misunderstood and avoid disclosing their symptoms for fear of being labeled as narcissistic or self-centered (1). Avoidance attitudes such as not participating in social activities and avoiding mirrors may also occur (1). People with BDD have symptoms similar to those of OCD, except for a lack of self-recognition of the mental illness and a poorer perspective (1). While most people experience some degree of dissatisfaction with their appearance at times, people with BDD experience persistent and intrusive thoughts about illusory flaws or defects in their appearance, especially regarding the nose, ears, mouth, and breast (1). Patients also obsess over their looks and body image, repeatedly checking the mirror, grooming, or seeking validation, sometimes for many hours per day (4, 5). Individuals suffering from BDD frequently focus attention on a body part to the point where it interferes with their social, emotional, educational, and occupational wellbeing (2, 4, 5).

BDD sufferers believe they have a genuine physical flaw and, as a result, seek cosmetic treatments to correct their perceived flaw rather than seeking mental health services to address their beliefs (3). They frequently visit dermatologists, beauticians, Botox clinicians, and cosmetic surgeons (3). Research and clinical experience show that people with BDD often feel deeply dissatisfied with the end results of such procedures and want additional physical cosmetic intervention, or their obsessiveness shifts to another aspect of their appearance (3).

BDD is believed to have its roots in psychological and physiological causes (2). BDD is thought to be caused by dysfunctional origins, such as abuse and, negative childhood experiences, and taunting, resulting in low self-esteem and insecurities (2, 6). People with BDD believe people taunt them or negatively comment about their appearance (5). They also possess tendencies toward perfectionism and constantly compare their appearance to others (5). Patients who had a distorted self-perception of body shape or a distorted comparative perception of body image were 3.67 or 5.93 times more likely to have severe BDD symptoms than those who had a more accurate perception (8) BDD is thought to be caused by dysfunctional origins, such as abuse and negative childhood experiences, and taunting, resulting in low self-esteem and insecurities (2, 6). Sixty-nine percent of BDD patients say they have been taunted or bullied at some point (8, 9). Now, as the social media trend grows, there is an extra, more relevant forum for bullying (2).

### Social media consumption and development of body dysmorphic disorder and tendencies toward cosmetic surgery

BDD and the widespread use of communication technology go in parallel. Based on existing data, frequent social media use may be a significant risk factor for BDD development (10). Khanna studied BDD cases in the Indian context and demonstrated the psychopathology of BDD that occurs through internet/mobile use (11). A case report addressed the impact of technology in activating and sustaining the psychopathology of BDD (11). In addition, body dissatisfaction among young women has been linked to social media use (12, 13). Studies in the UAE found that there is sufficient evidence in the literature to conclude that social media plays an essential role in shaping perceptions about body image and self-esteem (14). Researchers suggest social media exposure can lead to body dissatisfaction through various potential mechanisms, including comparisons and self-objectification (12).

Appearance-based comparisons were also shown to be a potent mediator of social media use and body image dissatisfaction (10). Women who repeatedly engage in comparisons with others are more prone to suffer compared to women who engage less in such comparisons (13). The negative comparison explains this effect by stating that women experience a comparison between themselves and thin, idealized models, which leads to lower body satisfaction (13). This causes issues for those with body image issues, particularly girls because society defines women more by their body image than men (11). This manifested in girls with BDD, who have inflexible, perfectionist attitudes about their appearance, resulting in poor self-evaluation and low self-esteem (11).

In addition, the behavioral model emphasizes expectancy theory and social learning in the development of body dysmorphic-related beliefs (15). The hypothesis was that participants who had a higher consumption using social media and photo-editing applications would tend to develop symptoms of BDD and have increased acceptance of cosmetic surgery compared with participants who had a lower consumption (15). A systematic search among young adults showed the unhealthy use of social media and appearance-focused tools in particular as a leading mediator for BDD (11). Furthermore, the systematic analysis found that appearance-based comparison is considered a determinantal factor in developing BDD symptoms (11). Further, according to the literature, passive social media use and appearance-focused social media use are incredibly influential in shaping the perception of one's body and thus, such consumption could be a risk factor for BDD development (10).

Social media amplified the visuals in our lives. Photographs and videos are involved in most popular apps (e.g., Snapchat and Instagram). As such, people have become more aware of their appearance with the trending use of selfies and filters (16). Social media provides limitless opportunities to present our best, often digitally altered and contrived self to the public (17). A study looked into the link between social media use in general, social media behaviors linked to taking “selfies” and sharing those selfies in particular, and body dissatisfaction and overvaluation of shape and weight (18). Girls who routinely shared self-images on social media had considerably higher overvaluation of shape and weight, higher body dissatisfaction, and took into consideration a unified idea of the thin ideal than those who did not share selfies frequently (18).

Previous research studied the impact of exposure to idealized thin bodies in commercials, publications, television, and music videos on young women's body image (13). Further, frequent selfie viewing is associated with lower self-esteem and life satisfaction, which may lead to body dysmorphic disorder (17). Research frequently demonstrated a link between exposure to modest ideals and body dysmorphia in women (13).

In the same vein, social media is progressively identified as a factor in the growing public interest in cosmetic procedures (19). The high prevalence of social media and photo-editing applications has a significant impact on cosmetic surgery (17). The number of people seeking cosmetic operations has increased dramatically in recent years (2). More than 400,000 Americans had cosmetic surgery in 1992 (2). However, in 2015, a total of 21 million cosmetic operations were performed globally, with 15.9 million in the United States (2).

In early 2018, several newspapers expressed concern about the adverse effects of social media platforms, like Snapchat and Instagram, on users' decisions to undergo plastic surgery (4, 20). These two platforms offer taking frontal pictures called selfies and built-in filters that allow users to change their skin tone, soften fine lines and wrinkles, change the size of their eyes, lips, and cheeks, and alter other aspects of their physical appearance before sharing self-images on social media (4, 20). A study findings suggest that using certain social media and photo editing apps may be associated with increased acceptance of cosmetic surgery (17). Additionally, in Saudi Arabia young individuals reported 5.8% desire to enhance their appearance and selfies was a reason why they underwent cosmetic surgery, and 38% wished to have cosmetic surgery to look better in selfies (21). Moreover, in recent studies, selfies are the leading cause of plastic surgery among young people (11). Literature found that filters enhance physical traits, which may drive people to seek cosmetic operations, as several plastic surgeons observed that their patients request to look like their filtered photographs (20). Plastic surgeons who have seen patients requesting to look like their filtered pictures have observed that the widespread availability of photo-editing tools can do more harm than good (20).

Snapchat filters instill unrealistic beauty standards in young girls, leading to low self-esteem and image problems (17). Because of the growing popularity of selfies and photo editing, cosmetic surgeons have coined the term “Snapchat dysmorphia” to describe the psychological phenomenon of patients bringing filtered selfies to their surgeons to demonstrate the surgical changes they want to achieve (17). This intrusive focus engenders copying and modeling behaviors to fit with beauty standards. Thus, the demand for cosmetic surgery has increased (16). It is not uncommon for patients with BDD to continually seek cosmetic treatments to improve their physical appearance, which could worsen their mental health (2, 6, 9). In addition, a study among 2,510 German individuals showed individuals with BDD reported significantly more often a history of cosmetic surgery compared to individuals without BDD (9). Another study surveyed young adult participants has found users of Snapchat and/or Snapchat photo filters showed a higher overall score on the Acceptance of Cosmetic Surgery Scale (18). Furthermore, a survey of Italian women found that using Instagram led to body dissatisfaction and appearance comparison when engaged in both self-images, which had a significant effect on acceptance of cosmetic surgery (22).

### Heavy consumption of photo editing apps is a risk factor for BDD

Research conducted in Australia and New Zealand found that increased use of social media platforms, particularly Instagram and Snapchat, was associated with increased body image concerns (12). One hundred forty-four females (14–18 years old) were randomly exposed to either original or altered (retouched and reshaped) Instagram selfies in a between-subject experiment (12). The findings revealed that more exposure to modified Instagram photos results in a negative body image (12). Also, exposure to the modified photographs had a negative impact on girls with stronger social comparison tendencies (13). Girls exposed to modified Instagram photographs have lower body satisfaction than those exposed to real photos (13). A survey of social media users in Saudi Arabia reported BDD in 4% of users, with a higher prevalence in young users (8). Users spending longer time on Snapchat and Instagram were significantly associated with a high probability of having BDD (8). Moreover, an interview of 18 active female users of Snapchat and Instagram at California State University has found participants believed social media and society have generated unrealistic beauty standards (23). These overwhelming standards caused them to feel insecure about their physical appearance (23).

### Sociodemographic characteristics of people having tendencies toward plastic surgery

Saudi Arabia has recently seen an increase in the popularity of plastic surgery (24, 25). As per the International Society of Aesthetic Plastic Surgeons (ISAPS), among the top 30 countries with the highest rates of cosmetic procedures in the world, Saudi Arabia ranks 29th (25). Cosmetic surgery for both men and women increased by 58% between 2012 and 2016 in Saudi samples (24, 25). As cosmetic surgery trends grow, more dermatologists and cosmetic surgeons will encounter patients with BDD in their practices (2). A study in Saudi Arabia assessed the impact of filtered images on seeking cosmetic surgery has found that 38% of the study sample thought selfies increased their desire to undergo cosmetic procedures, with 85% of them being females (20). A survey of Iranian medical science students indicated that higher BMI, females, and singlehood were factors predicting the tendency toward cosmetic surgery (26). Moreover, a systematic review revealed that social media impacts the acceptance of cosmetic surgery with women representing 90% of all cosmetic surgery patients. Different countries, cultures, and ethnicity varied on how to embrace cosmetic surgery (27).

The dramatic increase in social media consumption in the past decades comes in parallel with a host of mental health issues. A large body of research has examined the negative impact of extensive social media use on wellbeing. Some studies have linked body dysmorphia to the negative use of these social media. Social media seems to have the effect of normalizing standard beauty criteria, which affects one's thoughts about their own physical appearance and probably increases their acceptance of cosmetic surgery. In Saudi Arabia, the prevalence of BDD has only been studied in students, dermatology patients, and people undergoing facial plastic surgery (28, 29). More large-scale research must be conducted to have generalizable and representative findings. This project, the first of its kind and scale, aims to assess the relationship between social media consumption, especially Instagram and Snapchat, and BDD and attitudes toward cosmetic surgery. Given the pervasion of smartphones and the increasing trends of social media use, this research can shed light on the side effects of technology and how it insidiously interferes with our psyche and body. Findings from this study can help policymakers and civic organizations to advocate for better regulations and practices.

#### Research question

What is the association between social media use and development of body dysmorphic disorder and attitudes toward cosmetic surgeries among Saudis aged 18 and above?

#### Objectives

- Assessing the relationship between social media use, especially Instagram and Snapchat, and BDD and attitudes toward cosmetic surgery. Given among Saudis above 18 years?
- Measuring the prevalence of BDD among Saudis.
- Identifying the risk factors for BDD.
- Assessing the characteristics of people having tendencies toward plastic surgery.

## Methodology

### Study setting and data collection

This research was designed as a descriptive cross-sectional study among the Saudis. Data were collected between January to February 2022 through an online questionnaire using validated scales. The web-based electronic survey was established using Qualtrics. The survey was piloted by 20 persons considering both gender and variety in ages and educational level.

Data collection was done by distributing an online questionnaire via social media channels. We used various social media channels to distribute the survey, such as WhatsApp, LinkedIn, Telegram, and Twitter, on January 22, 2022. The questionnaire allowed for the collection of self-reported dysmorphic concerns and acceptance of cosmetic surgery attitudes that were required for this study.

### Population sample

Convenience sampling was used in this study. The sample size was determined using the adult population in Saudi Arabia with a confidence interval level of 95%, a 5% margin of error, and a 5% level of significance. The required estimated sample was approximately 400 Saudi adults.

The questionnaire was initially completed by 1,650 participants. Saudis aged 18 and above who used Snapchat and/or Instagram were included. One Sixty-Seven participants were not eligible because of age, nationality, or not using Snapchat and/or Instagram, so they were excluded. The total sample size was 1,483. All responders who met the eligibility criteria were included in the analysis. The demographic breakdown is detailed in the results chapter.

### Questionnaire design

The survey has three main sections: (1) sociodemographic data, (2) social media use and behaviors, (3) Dysmorphic Concern Questionnaire (DCQ) scale, and (4) Acceptance of Cosmetic Surgery Scale (ACSS).

Social media use and behaviors assessed via a 4-point Likert scale, which asks, for example: do you use filters when taking selfies? (Always, Sometimes, Rarely, Never).

DCQ: The DCQ is a seven-item self-report questionnaire that examines cognitive and behavioral features of dysmorphic concern. Responses evaluated on a four-point response scale ranging from 0 “Never” to 3 “All of the time”. All seven items of the DCQ summed up to result in one total score with no subscales. The total score may range from 0 to 21. Higher scores indicate higher levels of altitudinal disturbance regarding one's appearance. A DCQ score cut-off of 11 was selected to identify participants with BDD. This was based on the study where this cut-off showed good internal consistency with Cronbach's α = 0.81 (30). Overall, the DCQ has shown high internal consistency and structural validity (30, 31).

The Acceptance of Cosmetic Surgery Scale (ACSS) uses a five-point Likert scale (1= strongly disagree, 5= strongly agree) which assesses attitudes toward cosmetic surgery, and it consists of 15 items. Cosmetic surgery attitudes are based on three domains: (1) consideration, (2) social, and (3) intrapersonal. The overall score is calculated by adding the items' scores; therefore, the possible range is 15–75. The higher scores indicate higher acceptance of cosmetic surgery (17). ACSS provides evidence to have high internal consistency with Cronbach's α = 0.95, good test-retest reliability, and good convergent and divergent reliability (32, 33).

### Validity and reliability

Both scales were obtained in English and then translated into Arabic by a bilingual expert through two steps: translation and back translation, taking into consideration the cultural fit.

To determine the face and content validity of the scales, a pilot study with 20 adults was done. The whole questionnaire was sent in Arabic and English to target Arabic and non-Arabic speaking from different demographics. We asked them to provide their suggestions regarding the context, linguistics, ambiguity, and simplicity of the translated version of the questionnaire. The questionnaire was assessed to be clear and simple. Thus, no changes were made to the wording or substance of the questions. The validity of the questionnaire remained intact.

### Variables

The first section of the survey includes sociodemographic data: age, gender, whether or not using Snapchat and Instagram, nationality, marital status, economic status, and education level.

The second section includes three questions about social media use and behaviors assessed via a 4-point Likert scale, which asks, for example: Do you use filters when taking selfies? (Always, Sometimes, Rarely, Never). Also, a question to measure the exposure level to social media will be measured by the following question: “How much time do you usually spend on Instagram and Snapchat” (< 1 h a day = low exposure; 1–3 h a day = average exposure; 4–7 h or more = high exposure).

Focusing on body dysmorphia, the third part of the survey was about dysmorphic concerns; (DCQ) scale will be used to assess body dysmorphic disorder (BDD). DCQ uses four-point scale ranging from 0 “Never” to 3 “All of the time”. An example item is “Have you ever spent a lot of time covering up defects in your physical appearance or bodily functioning?”

The last part of the survey looked at the Acceptance of Cosmetic Surgery Scale (ACSS) uses a five-point Likert scale (1= strongly disagree, 5= strongly agree) which assesses attitudes toward cosmetic surgery, and it consists of 15 items. Cosmetic surgery attitudes are based on three domains: (1) consideration (5 items, e.g., the degree to which an individual would consider having cosmetic surgery); (2) social (5 items, e.g., acceptance of cosmetic surgery based on social motivation); and (3) intrapersonal (5 items, e.g., the acceptance of cosmetic surgery based on intrapersonal motivation).

### Ethical consideration

Ethical exemption from approval was guaranteed by the Institutional Review Board (IRB) at Alfaisal University (IRB Log Number: 20143 on May 25, 2023). The survey was established according to the guidelines of the Declaration of Helsinki. Participation in the study was voluntary, and withdrawal allowed at any time. The authors did not use any identifying data to ensure anonymity and confidentiality. Access to data was restricted to the authors only, and all responses were encoded. The authors used the data for research purposes only.

### Data analysis

The data entered in an M.S. Excel spreadsheet and the Statistical Package for Social Sciences (SPSS) version 21.0. used for analysis. Significance set at *p* < 0.05. Descriptive analyses conducted to describe the study sample and the prevalence of BDD, which presented as frequency and percentages. The analysis of the numerical variables (DCQ and ACSS scores) is given as mean and standard deviation. The scores for DCQ were found to be skewed to the right—the data were analyzed using both the parametric (ANOVA) as well as the non-parametric (Kruskall Wallis test). The results for both methods were found to be the same—due to the large sample size (standard error was minimized due to the large sample size). So, the numerical scores are reported as mean + standard deviation, and ANOVA was used to compare the mean scores. Tukeys *post-hoc* test was done to identify the categories that were different when the ANOVA test showed a statistically significant association. The prevalence of BDD was determined by taking a cutoff score of 11 and above for the DCQ score, and the 95% confidence interval was determined for the prevalence. The prevalence of BDD was compared between the categorical variables using the chi-square test. The ACSS mean score was compared for the prevalence of BDD using the Independent Samples *t*-test, which is shown as a box plot. The ACSS mean scores were compared between the categorical variables using ANOVA, followed by *post-hoc* test when necessary. Chi-square tests used to measure association between the main study variables. Also, ANOVA was used to assess mean differences in BDD and attitudes toward cosmetic surgeries based on heavy, average, and low social media consumption. Demographic variables were assessed between the two BDD categories using the Chi-Square test.

## Results

### Demographics

There was a total of 1,483 participants who responded to the online questionnaire. Table 1 indicates the demographic characteristics of the study population. The average age of the study participants was 18–40 years and above, 557 participants aged 18–24 years old (38%), 413 aged 30–39 years old (28%), 357 aged 25–29 years old, 156 aged 40 and above (10%). Of the respondents, 1,043 (70%) were females, and 440 (30%) were males. The majority of the participants were single, 901 (61%), married 530 (36%), and divorced/widow only 52 (3%). Academically, the survey's most prevalent responders were bachelor degree holders 1,004 (67%), Master's/PhD 230 (16%), High school or below 166 (11%), and diploma 83 (6%). Economically, the highest percentage of participants earned a good economic status 654 (44%), followed by 510 (34%) having a very good economic status, then an excellent economic status at 280 (19%). Low wages, reportedly as poor economic status, constitute only 39 (19%).

**Table 1:** Demographics of the respondents (*N* = 1,483).

		***N***	**%**
Gender	Male	440	30%
	Female	1,043	70%
Age (years)	18–24	557	38 %
	25–29	357	24%
	30–39	413	28 %
	40+	156	10%
Education	High school or below	166	11%
	Diploma	83	6%
	Bachelors	1,004	67%
	Master's/PhD	230	16%
Economic status	Poor	39	3%
	Good	510	34%
	Very good	654	44%
	Excellent	280	19%
Marital status	Single	901	61%
	Married	530	36%
	Divorced/Widowed	52	3%

### Social media practices of the respondents

Table 2 shows that, on average, the population spent 1–3 h a day on Instagram and Snapchat apps, constituting the highest percent, 749 (51%) among other respondents, while 392 (26%) spent 4–7 h daily. Moreover, 233 (16%) spend less than an hour daily on social media, while 109 (7%) spend more than 7 h daily. Out of 1,483, 1,347 (91%) consider the vast majority of the sample used selfie services with varied regularity (rarely, sometimes, always), whereas a small percentage have not used selfies 136 (9%). The most prevalent took selfies in the sample, sometimes 589 (40%) followed by 482 (32%) rarely used. However, 276 people (19%) have always taken selfies. Of 1,347 who took selfies, around 1,190 (88%) used a filter to modify the photos, while 157 (12%) did not. Selfie users who used filters always were 568 (42%), while those who used filters sometimes were 441 (33%), and those who used filters rarely were 181 (13%). Outside of 1,347, Only 126 (9%) of the 1,347 individuals who reported taking selfies did not share them. That being said, 1,221 (91%) had shared selfies with others, 290 of them (22%) stated they always share selfies, 561 (42%) shared selfies sometimes, and 370 (27%) have rarely been sharing selfies. Out of 1,221 participants reported they shared selfies through different social media platforms; the Result shows the vast majority of them posted selfies via Snapchat 1,102 (74%). WhatsApp and Instagram score 182 (12%), and 167 (11%), respectively. A few 84 (3%) posted selfies using other apps.

**Table 2:** Social media practices of the respondents.

		***N***	**%**
Time spent on Instagram and Snapchat (*N =* 1,483)	< 1 h a day	233	16%
	1–3 h a day	749	51%
	4–7 h a day	392	26%
	>7 h a day	109	7%
Take selfies (*N =* 1,483)	Never	136	9%
	Rarely	482	32%
	Sometimes	589	40%
	Always	276	19%
Use filters to edit selfies (*N =* 1,347)	Never	157	12%
	Rarely	181	13%
	Sometimes	441	33%
	Always	568	42%
Share selfies with others (*N =* 1,347)	Never	126	9%
	Rarely	370	27%
	Sometimes	561	42%
	Always	290	22%
Account used to post selfies—multiple options allowed (*N =* 1,221)	Snapchat	1,102	74%
	Instagram	167	11%
	WhatsApp	182	12%
	Others	84	3%

### Mean Dysmorphic Concern Questionnaire (DCQ) scores related to social media practices of the respondents

As summarized in Table 3, Result of viewing Dysmorphic Concern Questionnaire scores related to social media practices shows respondents who spent 4–7 h (M = 7.42, SD = 5.36) or more than 7 h a day (M = 7.06, SD = 5.35) on Instagram and Snapchat had significantly a higher Dysmorphic Concern score than those who spent < 1 h a day (M = 5.78, SD = 5.17) (*p* < 0.001). Taking selfies and Dysmorphic concern had a significant progressive intercorrelation (*p* < 0.001). Respondents who always use selfies (M = 7.36, SD = 5.57) and sometimes using selfies (M = 6.91, SD = 5.17) were more likely to have dysmorphic concerns than respondents who never use selfies (M = 5.18, SD = 4.66). Always frequency of using filter (M = 8.56, SD = 5.4) to edit photo were the most significant contributor to dramatic growth of persons's dysmorphic concern among other less frequent measures, sometimes (M = 5.84, SD = 4.86) and rarely (M = 4.86, SD = 4.38) and never (M = 4.69, SD = 4.74) (*p* < 0.001). Selfies shared with others had no significant influence on dysphoria (*p* = 0.07).

**Table 3:** Mean Dysmorphic Concern Questionnaire (DCQ) scores related to social media practices of the respondents.

		**DCQ mean score**				
		***N***	**Mean**	±	**SD**	***p*** **-value**
Time spent on Instagram and Snapchat (*N =* 1,483)	< 1 h a day	233	5.78	±	5.17	**< 0.001**
	1–3 h a day	749	6.31	±	5.12	
	4–7 h a day	392	7.42	±	5.36	
	>7 h a day	109	7.06	±	5.35	
Take a selfie (*N =* 1,483)	Never	136	5.18	±	4.66	**< 0.001**
	Rarely	482	6.12	±	5.17	
	Sometimes	589	6.91	±	5.17	
	Always	276	7.36	±	5.57	
Use filters to edit selfies (*N =* 1,347)	Never	157	4.69	±	4.74	**< 0.001**
	Rarely	181	4.86	±	4.38	
	Sometimes	441	5.84	±	4.86	
	Always	568	8.56	±	5.4	
Share selfies with others (*N =* 1,347)	Never	126	6.17	±	5.02	0.07
	Rarely	370	6.33	±	5.4	
	Sometimes	561	7.14	±	5.17	
	Always	290	6.64	±	5.36	

p-values determined using ANOVA and pair-wise comparisons using Tukey's HSD are categorized in bold.

DCQ, Dysmorphic Concern Questionnaire.

### Mean Acceptance of Cosmetic Surgery Scale (ACSS) scores related to social media practices of the respondents

As shown in Table 4, Looking for Acceptance of cosmetic surgery with relation to social media usage. Result shows no significant differences between individuals accepting cosmetic surgery and time spent on Instagram and Snapchat (*p* = 0.24). When compared to never using selfies (M = 1.33, SD = 0.97), using selfie services (M = 1.60, SD = 1.04) revealed to be a significant influence in accepting cosmetic surgery (*p* < 0.001). Furthermore, as compared to respondents who rarely use selfies (M = 1.60, SD = 1.04), respondents who always use selfies (M = 1.87, SD = 1.10) resulted in a significant difference in ACSS scores (*p* < 0.001). Additionally, the acceptance of cosmetic surgery significantly raised as the frequency of using filters in editing photos increased. Participants who never use filter (M = 1.34, SD = 0.89), and those who use filter rarely (M = 1.41, SD = 1.03), were remarkably similar, but in comparison to sometimes users (M = 1.61, SD = 0.97), a significant difference was discovered (*p* < 0.001). In addition, participants who always use filters (M = 2.00, SD = 1.08) significantly had higher mean ACSS score than those who sometimes use filters (M = 1.61, SD = 0.97) (*p* < 0.001). Accepting cosmetic surgery score (ACSS) was influenced by the social media practice of sharing selfies with others. It was seen that persons who never shared their selfies with others had a lower ACSS mean score (1.39 ± 0.98) as compared to those who shared selfies rarely (1.67 ± 1.02), sometimes (1.75 ± 1.04), or always (1.84 ± 112) (*p* = 0.001).

**Table 4:** Mean Acceptance of Cosmetic Surgery Scale (ACSS) scores related to social media practices of the respondents.

		**ACSS mean score**				
		***N***	**Mean**	±	**SD**	***p*** **-value**
Time spent on Instagram and Snapchat (*N =* 1,483)	< 1 h a day	233	1.59	±	0.99	0.24
	1–3 h a day	749	1.69	±	1.04	
	4–7 h a day	392	1.68	±	1.10	
	>7 h a day	109	1.83	±	1.04	
Take a selfie (*N =* 1,483)	Never	136	1.33	±	0.97	**< 0.001**
	Rarely	482	1.60	±	1.04	
	Sometimes	589	1.74	±	1.02	
	Always	276	1.87	±	1.10	
Use filters to edit selfies (*N =* 1,347)	Never	157	1.34	±	0.89	**< 0.001**
	Rarely	181	1.41	±	1.03	
	Sometimes	441	1.61	±	0.97	
	Always	568	2.00	±	1.08	
Share selfies with others (*N =* 1,347)	Never	126	1.39	±	0.98	**0.001**
	Rarely	370	1.67	±	1.02	
	Sometimes	561	1.75	±	1.04	
	Always	290	1.84	±	1.12	

p-values Determined Using ANOVA and Pair-Wise Comparisons Using Tukey's HSD are categorized in bold. ACSS, Acceptance Cosmetic Surgery Survey.

### Measuring the prevalence of BDD among Saudis

The prevalence of BDD (DCQ score of 11 and above) is 24.4% (95% CI: 22.2%, 26.6%).

### Comparison of prevalence of BDD by demographic characteristics

Body dysmorphic disorder (DCQ score of 11 and higher) was found in 24.4 % of Saudis. As summarized in Table 5, females (29%) were significantly having almost twice the opportunity to be affected by BDD as males (15%) (*p* < 0.001). The prevalence of BDD was significantly influenced by age, with the highest proportion (30%) reported in the 25–29-year-old group, followed by (24%) in the 18–24-year-old group, (22%) in the 30–39-year-old group, (19%) in the 40-years-old group (*p* = 0.013).

**Table 5:** Comparison of prevalence of BDD by demographic characteristics.

		**BDD**				
		**Normal (DCQ**<**11)**		**BDD (DCQ 11**+**)**		
		***N***	**%**	***N***	**%**	***p*** **-value**
Gender	Male	376	85%	64	15%	**< 0.001**
	Female	745	71%	298	29%	
Age	18–24	423	76%	134	24%	**0.013**
	25–29	249	70%	108	30%	
	30–39	322	78%	91	22%	
	40+	127	81%	29	19%	
Education	High school or below	130	78%	36	22%	0.75
	Diploma	65	78%	18	22%	
	Bachelors	753	75%	251	25%	
	Master's/PhD	173	75%	57	25%	
Economic status	Poor	22	56%	17	44%	**< 0.001**
	Good	385	75%	125	25%	
	Very good	481	74%	173	26%	
	Excellent	233	83%	47	17%	
Marital status	Single	660	73%	241	27%	**0.025**
	Married	422	80%	108	20%	
	Divorced/Widowed	39	75%	13	25%	

p-values determined using Chi-Square test are categorized in bold.

BDD, Body Dysmorphic Disorder.

DCQ, Dysmorphic Concern Questionnaire.

Saudis with poor economic status (44%) had significantly double the risk of being affected by BDD compared to Saudis with excellent economic status (17%) (*p* < 0.001). Unlike economic status, educational degree level high school or below (22%), diploma (22%), bachelor's (25%), master's/PhD (25%) had no statistically significant effect on BDD prevalence (*p* = 0.75). Married women had significantly lower BDD (20%), as compared to those who were Single (27%), or Divorced/Widowed (25%) (*p* = 0.025).

### ACSS mean score comparison for BDD prevalence

**Figure 1:**
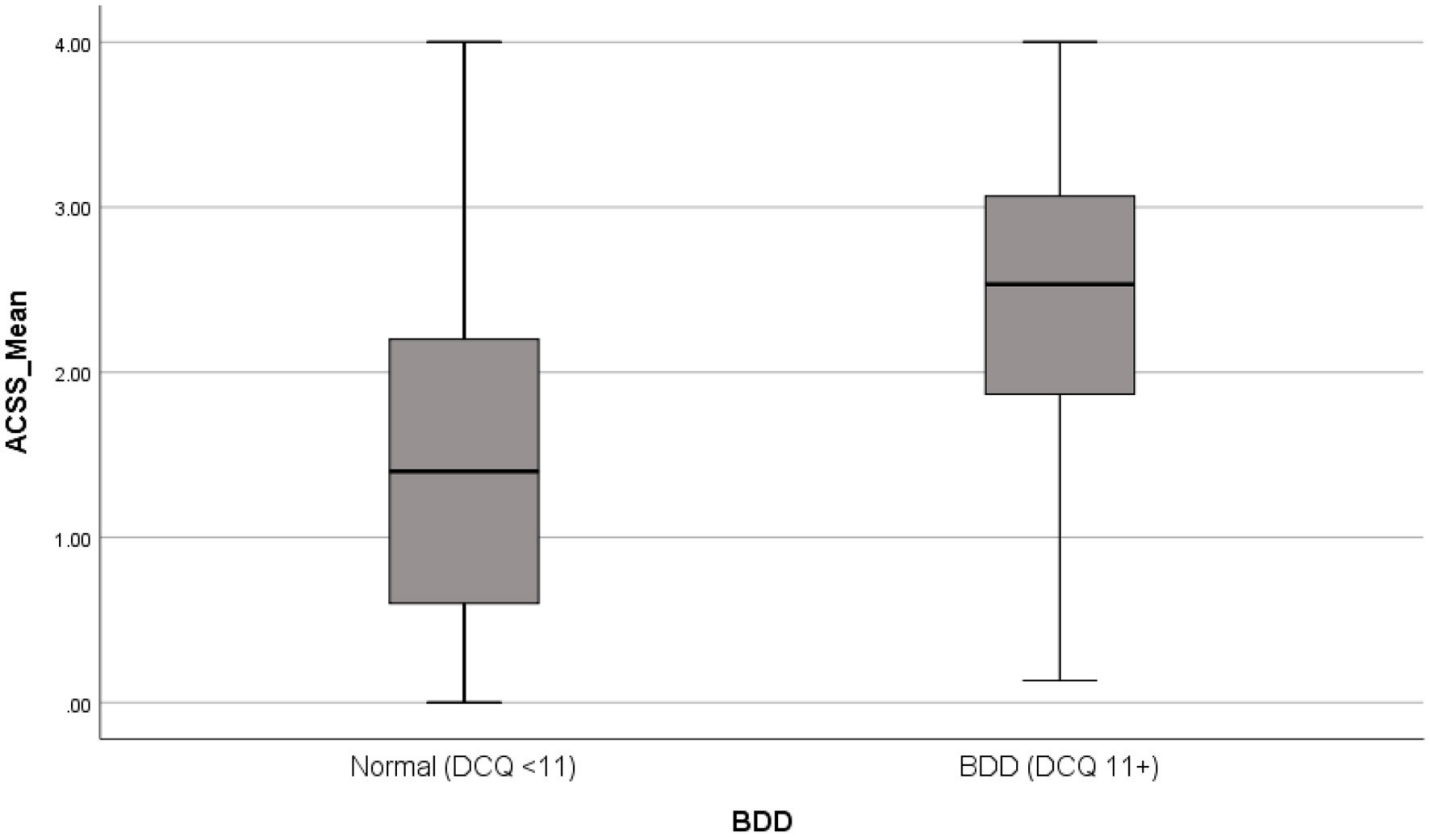
ACSS mean score comparison for BDD prevalence. ACSS, acceptance cosmetic surgery survey; BDD, body dysmorphic disorder.

Individuals with Body Dysmorphic Disorder had a significantly higher risk of accepting cosmetic surgery (*p* < 0.001), as indicated in Figure 1.

### Comparison of mean ACSS scores by demographic characteristics

According to findings in Table 6, gender is a major factor in raising the likelihood of accepting cosmetic surgery with female respondents (M= 1.74, SD = 1.05) outnumbering male respondents (M = 7.06, SD = 5.35) (*p* < 0.001). Aging significantly shows a greater concern of cosmetic surgery acceptance among respondents (*p* < 0.001). The 18–24 years age group (M = 1.41, SD = 1.00) had much lower acceptance cosmetic surgery attitude in compared to 40+ years age group (M = 2.02, SD = 1.14), 30–39 years age group (M = 1.84, SD = 1.00), 25–29 years age group (M = 1.77, SD = 1.05). There was significant low concern about accepting cosmetic surgery at the high school or below level (M = 1.48, SD = 0.95), compared to diploma (M = 1.82, SD = 1.85), and master/PhD educational level (M = 1.85, SD = 1.03) (*p* = 0.003). Cosmetic surgery acceptance attitude was found to be strongly driven by economic status (*p* = 0.03), Saudis with excellent status had low interest (M = 1.54, SD = 1.09) and poor Saudis had higher interest (M = 2.01, SD = 1.12). Divorced/Widowed Saudis acceptance cosmetic surgery attitude (M = 1.96, SD = 0.79), varied significantly in contrast to Saudi singles (M = 1.59, SD = 1.03) (*p* < 0.001).

**Table 6:** Comparison of mean ACSS scores by demographic characteristics.

			**ACSS mean score**			
		***N***	**Mean**	±	**SD**	***p*** **-value**
Gender	Male	440	1.55	±	1.02	**0.001**
	Female	1,043	1.74	±	1.05	
Age groups	18–24 yrs	557	1.41	±	1.00	**< 0.001**
	25–29 yrs	357	1.77	±	1.05	
	30–39 yrs	413	1.84	±	1.00	
	40+ yrs	156	2.02	±	1.14	
Highest educational degree	High school or below	166	1.48	±	0.95	**0.003**
	Diploma	83	1.82	±	0.97	
	Bachelors	1,004	1.66	±	1.07	
	Masters/PhD	230	1.85	±	1.03	
Economic status	Poor	39	2.01	±	1.12	**0.03**
	Good	510	1.70	±	1.00	
	Very good	654	1.71	±	1.06	
	Excellent	280	1.54	±	1.09	
Marital status	Single	901	1.59	±	1.03	**< 0.001**
	Married	530	1.80	±	1.07	
	Divorced/Widowed	52	1.96	±	0.97	

Significant p-values determined using chi-square test are categorized in bold.

ACSS, Acceptance Cosmetic Surgery Survey.

## Discussion

### Social media consumption and development of body dysmorphic disorder and tendencies toward cosmetic surgery

Body dysmorphic disorder is a common psychiatric illness and often severe (34, 35). To the best of our knowledge, this is the first study that examines the association between social media use and BDD and attitudes toward cosmetic surgery in Saudi Arabia. Also, this study measured the prevalence of BDD among Saudis, identified risk factors for BDD, and assessed the sociodemographic characteristics associated with tendencies toward plastic surgery.

Findings supported our first hypothesis by demonstrating that higher social media consumption and frequent use of selfies and filters on Instagram and Snapchat were associated with heightened concerns about body image, resulting in increased acceptance of cosmetic surgery. These findings are aligned well with the Social Cognitive Theory (SCT), which asserts that expectations and norms shape human behaviors and attitudes. Social expectations and standards about body image, often portrayed through social media, influence how one develops a vision of the “ideal” body image, and therefore seeks cosmetic surgery to fit in with such standards. Images of models and enhanced portraits promulgated via social media have created the criteria for a sociocultural model of beauty. Individuals, then, internalize such beauty criteria and adopt others' perceptions of beauty as their own. A meta-analytic review shows a strong association between the use of social media and the internalization of the ideal image (36). By design, social media amplifies the effect of these social norms as these platforms put overwhelming emphasis on physical appearance and visuals, which then engenders body-related comparison, body surveillance, and attention bias toward body features.

Body-related comparison is a social comparison and often causes concerns about body image. It is the process when one compares their own body with those of others. This process can be self-initiated by people judging themselves or externally triggered by the environment when a person is exposed to judgment to resemble the ideal standards of beauty. This social comparison results in pressure, and it reinforces one's thoughts about their physical appearance, which explains some of the influence of the media on the image. Previous research suggests that body image concerns correlate with higher reported social comparison regarding physical appearance (37). The amount of appearance-related social media photo activity is linked to higher levels of internalization and appearance comparison (38). As a result, users adopt a standard definition of “attractive” appearance and then engage in modeling behavior to fit in. Overall, regular exposure to sociocultural criteria of beauty through social media stimulates the likelihood of overthinking of one's appearance.

Social media can affect body image in different ways. The design of apps, like Snapchat and Instagram, allows individuals to capture a frontal picture of their own faces (i.e., selfies) and to also edit these photos with an array of beautifying filters. Most filters alter facial features in certain ways to beautify one's appearance, often in an unrealistic way. Such features can cause continuous feelings of dissatisfaction about one's body. A systematic review found concerns about physical appearance increased as a result of editing selfies (39). Social media users focus more on their appearance when participating in photo editing apps activities, which subsequently increases the internalization of unrealistic beauty (38). It can be derived that it is likely that frequently engaging with selfies and filters may encourage hyperattention on unrealistic beauty standards and desires to change one's physical appearance.

Social media pressures for certain criteria of beauty and the internalization of cultural appearance ideals consistently emerge as predictors for BDD. Our findings are in agreement with a meta-analysis study that shows exposure to the thin ideal image portrayed in magazines and on television has been linked to body dissatisfaction (36). The new trends in photo-editing apps on social media centered on unrealistic beauty can be a risk factor to trigger the prevalence of BDD. Although our findings reported that taking selfies increases the likelihood of developing BDD while posting selfies does not, an experimental study comparing the selfie-takers group to the control group indicates that sharing selfies on social media can have a negative psychological impact (40). Furthermore, a study by Khanna (2017) stresses the role of selfie technology in triggering and maintaining the psychiatric symptoms of BDD and the importance of screening selfie use as a comorbid condition in cases of BDD (12). In general, as proven by literature social media emphasizes certain beauty criteria, which place pressure to conform to these beauty standards, perpetuate negative thoughts about an individual's body, and subsequently can engender BDD (41).

Having BDD is considered a substantial risk factor in developing thoughts about cosmetic surgery. Findings from our study are congruent with a study that reveal that regular use of social media apps by UK and US residents predicts body dissatisfaction and openness to cosmetic surgery (42). Another study confirms that a higher tendency to consider cosmetic surgery is associated with spending more time on social media platforms (43). Moreover, several studies have found that selfies shape the expectation that the face should feature a “beautified” look, which may lead people to be more open to cosmetic surgery (15, 37, 44). A study in Italy found that body dissatisfaction results from Instagram apps significantly affects acceptance of cosmetic surgery (22). Moreover, several studies in China, the US, and Saudi Arabia found that selfies and regular use of filters influence accepting of cosmetic surgery (12, 18, 21, 45). Similarly, a survey among Saudi citizens indicated that sharing filtered selfies may be a factor when considering having cosmetic surgery (25). Further, research shows that Snapchat dysmorphia may result in body dysmorphic disorder (6, 23, 46). The term “Snapchat dysmorphia” refers to the phenomenon of patients asking for operations to look like their selfies or filtered pictures (47). Individuals with BDD disorder may draw inspiration for cosmetic surgeries from Snapchat and Instagram filters (6, 23, 48). Our study, in accordance with previous literature, concludes that attitude toward cosmetic surgery is associated with concerns about body appearance caused by selfies and filters (49, 50). As such, our conclusion shows that social media and photo editing features affect one's perception of oneself, which triggers BDD, and may cause higher acceptance of cosmetic surgery; thus, our first hypothesis was supported by evidence.

### Measuring the prevalence of BDD among Saudis

Regarding our second hypothesis, findings from this study reveal that self-reported BDD prevalence in Saudi Arabia is 24%. The reported rate is substantially greater than the 8.8% prevalence rate reported in another study conducted with a sample from a major Saudi Arabian city (51). It seems plausible that this variation may be due to the larger sample size of our study score as we collected data from different regions in Saudi Arabia. Globally, BDD affects 1.9 to 2.2% of people worldwide (52). The disparity between the prevalence rates of BDD among Arab and Western cultures may be due to sociocultural influences on body image. Also, differences in BDD prevalence across studies can be attributed to the lack of a unified screening method. Nevertheless, BDD is a shame-related disorder, so anonymized self-assessment tools may help provoke honest responses from our participants—potentially even more honest than personal clinical interviews. Given the widespread use of social media in Saudi Arabia and the established association of these platforms with BDD, it stands reasonable that the prevalence of BDD is in the higher end. Future studies, which include a structured diagnostic interview, would be necessary to confirm the diagnosis and prevalence rates of BDD, especially given that concerns about one's appearance are relatively regular in the community.

### Heavy consumption of photo editing apps is a risk factor for BDD

Our third hypothesis suggested that heavy consumption of photo editing apps like Snapchat and Instagram is a risk factor for BDD. The high penetration of the internet and easy access to social media platforms raise the consumption of these apps. Snapchat and Instagram can aggravate negative body image beliefs by frequently using selfies and filter services that promote unrealistic beauty. Thus, as supported by our findings, easy access and heavy use of selfies and filters can be a risk factor for BDD and can heighten the probability of its occurrence. Former studies found that individuals who report frequent interactions with filtered images show signs of BDD (48, 53, 54). Additionally, a large body of research found that persistent social media consumption leads to a detrimental psychological impact and generates dissatisfaction and negative thoughts about participants' body image (46, 54, 55). Although those who spent more than 7 h on Instagram/Snapchat showed no significantly low BDD mean score compared to those with 4–7 h of use, this difference might be attributed to users spending more than 7 h chatting, watching reels of fashion, home decor, and cooking. Overall, heavy users of photo-editing apps, especially Snapchat and Instagram, are prone to develop BDD. Hence, our third hypothesis was fully supported as heavy unhealthy consumption of social media apps is a risk factor for higher BDD prevalence.

### Sociodemographic characteristics of people having tendencies toward plastic surgery

Our result for the fourth hypothesis shows that BDD is more prevalent among young, single, poor, and female Saudis. This finding is consistent with a study that found BDD higher in females and single (54). This is in contrast to survey findings in Mekkah city, which reported no significant differences in age and gender between BDD and non-BDD groups (56). However, our research and much other research report gender as a contributing factor in developing BDD and ACSS (12). A survey among American people demonstrated gender differences in the prevalence of BDD, with women reporting greater rates of the disorder than males (57). It is known that females are more prone to the development of BDD because they have strict and predefined beliefs about how they should look, which results in a perpetuating self-assessment and low self-esteem (12, 54, 58). In addition, women are more exposed to social norms of beauty standards and are subject to more scrutiny about their appearances (37, 48, 59). A study found that in order to blend in and meet society's beauty standards, women edit their images and use beauty filters, and they change how they present themselves to fit in (45). Therefore, women score substantially higher on the cosmetic attitudes scale than males do. A study by Morait and many others reported that gender is a main predictor for BDD, with women having a higher prevalence and a greater propensity toward cosmetic surgery (27, 32, 47, 58, 60–62). In sum, it can be deduced that gender is one of the prominent risk factors in developing BDD and acceptance of cosmetic surgery.

### Strength of the study

First study in Saudi that delivers approximate understanding how social media use link to BDD and attitude of cosmetic surgery with emphasis on the new trend of features of the most common used photo editing apps (Snapchat and Instagram). It adds to the literature, especially given how social media is pervasive in this region of the world which goes in parallel with high prevalence and inescapable trend of cosmetic surgery.

Large sample size from all regions of Saudi yields estimates that better approximate the population parameters and produce more precise and representative results.

Use of validated comprehensive scales which proven from literature to be reliable, robust instruments studied to measure behaviors, attitude, and hypothesis we anticipate to exist. At that, culture fit integrity considered when translating by bilingual expert through two steps: translation and back translation. Additionally, face validity have been assured by piloting the scales among 20 adults who speak both Arabic and English which showed the questionnaire remain intact.

### Limitations of the study

The results of this study should be considered with limitations. First, it is a cross-sectional study, which prevents any conclusion about causality between the study variables. However, experimental studies have shown causality between social media and BDD. A recent systematic review of 43 experimental studies indicates that exposure to beauty standards in social media leads to increased body image concerns (47, 63). Furthermore, as per a study conducted in a psychiatric setting, frequent selfie use has been associated with appearance concerns (12).

The second limitation of our study involves the convenience sampling used, which might have caused the gender bias (70% female participation). Nonetheless, we ensured the sample had a good representation of all regions in Saudi Arabia. Also, recruitment was achieved via an array of channels. In particular, we used WhatsApp to reach all demographic groups, as WhatsApp in Saudi Arabia is the primary mode of communication for the overwhelming majority of men, women, and older and younger populations. Additionally, the survey was conducted with a much higher sample than the recommended calculated power. Future studies might strive to recruit more men in their samples.

Another limitation is inherent in cross-sectional studies that involve self-reported responses, which might affect the accuracy of responses. We make sure to avoid suggestive language and any leading questions. In addition, we have delivered the survey anonymously through the web to avoid observers' bias and to increase confidentiality and privacy. We also ensured that the survey was short to avoid responder bias due to fatigue.

Moreover, this study did not perform regression analysis. Multiple regression will be implemented to reach a more nuanced understanding of the association examined. The future version of this manuscript will show such analyses.

## Conclusion and recommendation

### Conclusion

A growing body of evidence suggests that social media may impact mental health in different ways. a systematic review and much other research has found social media platforms have a deleterious impact on their users (64, 65). Our study sheds lights on how consumption of these platforms is associated with negative appraisals about one's physical appearance and one's tendency toward cosmetic surgery. Findings from our research align well with other studies (66). We find a potential role for social media in propagating body dysmorphic disorder and, subsequently, propensity toward cosmetic surgery, especially among females and singles. The mechanism of such an effect probably involves perpetual social comparisons and the unrealistic beautified appearances that result from photo-editing filters in Snapchat and Instagram.

As a result, screening measures that assess clients' motivations for cosmetic surgery and psychological functioning can be implemented in cosmetic surgery clinics. If the motivation is to resemble photos seen on social media, cosmetic treatments are unlikely to produce positive results (2). Implementation of successful diagnostic clinical interviews would contribute to better identification of patients who will likely benefit the most from the procedure and avoid patients from being subjected to unnecessary procedures that may come with physical and economic costs.

Given how invasive social media is, this line of research needs to be further developed to help policymakers and private sectors with important insights in order to foster a body-positive environment that can allow people to communicate in a healthier way.

### Recommendations

#### Individual level

The market for cosmetic operations is profitable and thriving. The industry supports unrealistic constraints on beauty, which could be damaging to the body. Legal inspection and ethical considerations in a clinical setting might be necessary to protect patients, minimize invasive operations, and lower unnecessary costs.

#### Community level

Recently, cosmetic surgery has become more fashionable. Identifying the factors associated with increased BDD prevalence and heightened levels of cosmetic surgery would help to design awareness initiatives through social media channels. Corporate social responsibility can be a great enabler to support such campaigns.

#### Research level

Given the uprising phenomenon of social media, we recommend that future research follows the RCT design to allow for causality, as most of the existing studies are cross-sectional. There are many moderators, mediators, and confounders that can influence the association between social media use and BDD, so more RCTs and structure modeling analyses could paint a more accurate picture of this association. In addition, most research has been done on the younger population; however, in Saudi Arabia and many other countries, older populations make a significant portion of the census, and many of them use social media as well, so we recommend having research dedicated to understanding the psychology of cosmetic surgery among the aging population. Third, we used a validated scale, but another researcher might be interested in developing a culturally sensitive scale for Middle Eastern Arabs or Muslims as they may have different views of cosmetic surgery due to the different sociocultural backgrounds.

#### Policymaker level

At the policy level, governments should intervene to regulate content guidelines and to support healthier consumption of such pervasive platforms. Also, the private sector, pressured by legal bodies, can initiate an ethical commission to govern the digital environment in a way that restricts potential harms and promotes more ethical practices. Social media platforms are not disappearing anytime soon; thus, there is an immense need to improve their role in society.

Snapchat dysmorphia has been a continuously increasing phenomenon with negative health outcomes. The evidence supports the call to consider it as a clinical disorder in order to allocate resources and amend current protocols. The practice could change that mental health screening is conducted before performing any cosmetic procedure to help assess BDD cases.

## Data availability statement

The raw data supporting the conclusions of this article will be made available by the authors, without undue reservation.

## Ethics statement

Ethical review and approval was not required for the study on human participants in accordance with the local legislation and institutional requirements. Written informed consent from the participants was not required to participate in this study in accordance with the national legislation and the institutional requirements.

## Author contributions

KA: Writing—review & editing, Conceptualization, Data curation, Formal analysis, Funding acquisition, Investigation, Methodology, Project administration, Resources, Software, Validation, Visualization, Writing—original draft. MA: Writing—review & editing, Supervision. NA: Supervision, Writing—review & editing.
